# 

**Published:** 1856-09

**Authors:** 


### INDEX TO VOLUME II.

PAGE.
Æsthetics of Medicine, 50
An Address before the Graduating Class of Atlanta Medical College, 63
A Thesis on Acute Inflammation, 129
Abuse of Remedial Agents, 145
A Chapter Omitted, 189
Annual Address, 205
Atlanta Medical Society, 246
Atlanta Medical College, 303, 559, 632, 756
A Monograph on Ovarian Tumors, 407, 523
Aphorisms on Hygene and Nursing, 419, 481, 611
American Medical Association, 502, 504, 654
A Convention of Medical Editors, 506
An Address Introductory, &c., 577
A case of Excision of Hip Joint, 625
A Trip to Tennessee, 631
Address of K. R. Winston, 636
An Address in Atlanta Medical College, 641
A Review and Medical Testimony, &c., 675
Alum in Croup, 7[illegible]3
A case of Hypertrophy, 705
Asthma, 708
A Batch of Interesting Questions, 760
Amputation of Cervix Uteri, 765
Billious and Yellow Fever, 82
Bibliographical, 700, 360, 251, 185
Back Numbers, 376
Books, &c., received, 635
Belladonna, 766
Case of Catalepsy, 3
Congenital Hydrocephalus, 19
Case of Cephalocele, 47
Close of Lectures, 52
Cure of Itch, 124
Causes of Diseases, 141
Case of Fever, 202
Case of Infantile Epilepsy, 233
Contributions to Physiology of Heart, &c., 296
Case of Hon. Chas. Sumner, 372
Contest between, &c., 509
Cogitations, &c., 547
Controvertional, 568
Circular of Savannah Medical College, 569
Case of Colica Pictonum, 702
Cure of Ves. Vag. Fis., 703
Convulsions, 724
Criminal Abortions, 733, 760
Commencement, &c., 762
Dr. Fell's Cure, &c., 763
Death from Amyline, 704
Dr. Kane, 640
Dr. Livingston, 508
Dr. Arnott, 448
Dr. James McClintock, 764
Diseases of Atlanta, 198
Death of Dr. Scarlett, 184
Draper's Physiology, 128
Dry Gangrene, 1
Extraction of Needles, &c., 108
Essay on the Physiology, &c., 116
Editorial Correspondence, 432, 363, 241, 192
Etherization, 255
Errata, 256
Effervescing Powders, 269
Experimental, &c., 275
Ergot of Wheat, 302
Effect of Belladonna, &c., 300
Effect of Carbonic Acid, 320
Excerpts, &c, 395
Essay, 463
Excito-Secretory, &c., 769
Effects of Chloroform, 507
Extracts, &c., 553
Embalmment, 740
Foreign Correspondence, 513, 591, 449
Fœticide, 247
Female Medical Education, 26, 179
Flint on Kidneys, 188

PAGE.
Gelseminum Sempervirens, 765
Gaseous Colic, 17
Glycerine in Phthisis, 638
Hydrocele, 637
Homœopathy, 40, 193, 327
Hints to Diagnosis, 96
Influence of the Sun, 126
Influence of the Climate of Peru, 229
Introductory Lectures, 307
Invasion of the Toe-Nail, 447
Ill Effects of Quinine, 638
Iodine in Neuralgia, 764
Iodine, 114
Jefferson Medical College, 763
Kentucky Giantess, 703
Letter from Boston, 472
Leeches, 511
Letter from New York, 257
Laceration of Cervix Uteri, 110
Medical Society, 239
Medical Miscellanies, 259
Mode of Testing Hydrocele, 255
Menstruation in old Age, 189
Moral Insanity, 470
Medical Society of Georgia, 503
Medical College of South Carolina, 504
Meterological, &c., 766, 640, 704, 448, 389, 512
National Hotel Disease, 763
New Cure for Intermittent Fever, 769
Next Class, 576
Number of Matriculants, 55
Observations upon Menstruation, 10
Of the cure of Arthritis, &c., 31
On the use, &c., of Chemical Baths, 37
Operation for Hare-Lip, 72
On Small Pox, 76
Observations, &c., on Medical Plants, 112
On Lactate of Zinc in Epilepsy, 122
On Chantharidian, &c, 126
On Neuralgia, 215
On Anæsthetic Agents, 174
On Quinine, 494
On Inflammation of Uterus, 291
On Evil Effects of Marriage, 351
On "Phantom Tumors," &c., 683
Of Sudden Deaths, &c., 687
On Medical Properties, &c., of Chlorate Potassa, 517
Obstetric Notes. &c., 570
On use of Chloroform, 543
Obstetrical Memoranda, 579
Preparatory Medical School, 761
Proceedings, &c., of Medical Society of Georgia, 671
Prof. Campbell's Claim, 574
Prospects of N. C. Journal, 446
Physiology, 375
Prospects &c., of our Journal, 383
Prospects of the N. C. Journal, 387
Puerperal Fever, 148
Pompion Seed, 73
Politics and Medicine, 102
Pennsylvania College of Medicine, 55
Questionable Advertisements, 692
Removal, &c., of Gun Barrel, 520
Remarks upon Elephantiasis, 647
Remarks, &c., upon Veratrum Viride, 399
Report of Case of Monstrosity, 321
Report, &c., of Dislocation of Knee, 323
Report from Case Book, &c., 333
Receipts, 256, 383
Remarks upon Medical Plants, &c., 283
Review of Dr. N. S. David's Address, 286
Report of Hemorrhage from the Lungs, 467
Researches or Cause, &c., of the Blood, 511
Remarks on Vesico-Vag. Fistula, 172
Reminiscences of Dr. Physic, &c., 222
Remarks, &c., of Intermittent Fever, 105
Regimen, 124
Report of Gun-shot Wound, &c., 15

PAGE.
Reports, &c., received, ... 54
Record of Medical Science, ... 55
Stricture of the Rectum, ...113
Subscriptions Received, ...767, 512, 128
Severe Headache from Carious Teeth, ...201
Spinder's Chalk Ointment, &c., ...256
Scarlet Fever, ...257
Sketch of Geology of Tennessee, ...478
Supposed case of Congestion, &c., ...651
Somnambulism, &c., ...694
Savannah Medical College, ...632
Singular Case of Triplets, ...638
Sudden Deaths in Puerperal State, &c., ...727
Society of Alumni, ...757
The Family Physician, ...750
The American Medical Association, &c., ...757
The Editor of American Med. Gazette, ...761
To our Patrons, ...762
Treatment of Whooping Cough, &c., ...765
Translations, &c., ...623
The late John Norcum, ...385
Two cases of Typhoid, &c., ...391
The Syphilization of Children, ...338

PAGE.
The College Edifice, &c., ...374
The present No. of Atlanta Journal, ...376
The accessibility, &c., to Correspondents, ...376
The Little Sister, ...565
Tragic end of John Hunter, ...382
The Presidency, &c., of American Med. Asso. ...489
The Med. Test'y, &c., of Willard Parker, &c., ...491
The Next Session, ...502
The Abnormal Conditions of K dneys, ...158
To Subscribers, ...183
The Sydenham Society, &c., ...253
Third Course of Lectures, ...175
To our Patrons, ...176
The Abortion of Trade, ...128
The Effects of Dentition, &c., ... 43
University of Nashville, ...535. 445
University of Louisville, ...762
Veratrum Viride, ...757, 764
Vesico-Vag. Fistula, ...765
When is a Medical Teacher, &c., Superanuated, 511
What is Hereditary Transmissions, ...136
What is the Matter, ...336
William Lawrence, ...639

### SUBSCRIPTIONS RECEIVED.

A. Clements, Ga.; R. Dixon, Ga.; W. Somerville, Va.; G. W. McDowell, Ga.; H. W. Caffy, Ala.; C. P. Brown, Ga.; T. S. Denny, Ga., 2d Vol.; Dabney & Brunson, Tenn., 2d Vol.; J. H. Harrison, Ga., 2d Vol.; L. F. Duckett, S. C., 2d Vol.; J. J. Scott, S. C., 2d Vol.; J. A. Griffith, Ga., 2d Vol.

Congenital Hydrocephalus,........ 19

Female Education,........ 26

Of the possible cure of Suppurative Arthritis, with the preservation of mobility, 31

On the Use and Abuse of Chemical Baths,........ 37

Homœopathy,........ 40

The Effects of Dentition on Nursing Children,........ 43

Case of Congenital Cephalocele,........ 47

Æsthetics of Medicine,........ 50

### EDITORIAL AND MISCELLANEOUS.

Close of Lectures,........ 52

Reports and Announcements Received,........ 54

New Orleans School of Medicine,........ 55

Pennsylvania College of Medicine,........ 5

Number of Matriculates in the Atlanta Medical College,........ 55

Appointment of L. J. Robert, M.D., to the Chair of Physiology and Pathology of the Oglethorpe Medical College at Savannah, Georgia,........ 56

Record of Medical Science,........ 56

---

### TO CORRESPONDENTS.

☞ All Communications pertaining to the Editorial Department of the Journal, should be addressed to the Editors.

☞ All Communications, having reference to the Subscription, the Advertising or the Financial Department, should be addressed to the Treasurer, J. G. WESTMORELAND, M. D., Dean of the Faculty of the Atlanta Medical College.

---

☞ Messrs. A. TILDEN and W. H. PHILLPOT are authorized Agents for this Journal.

